# Cement Viscosity and Application Time Lead to Significant Changes in Cement Penetration and Contact Surface Area

**DOI:** 10.1016/j.artd.2024.101476

**Published:** 2024-10-17

**Authors:** Christian Fölsch, Julia Schirmer, Cosmin Glameanu, Bernd Ishaque, Carlos Alfonso Fonseca Ulloa, Torben Harz, Markus Rickert, John Ryan Martin, Jan Scherberich, Jessica Steinbart, Gabriele Krombach, Christian Paul, Klaus-Dieter Kühn, Alexander Jahnke

**Affiliations:** a Department of Orthopaedics and Orthopaedic Surgery, University Hospital Gießen and Marburg (UKGM), Justus-Liebig-University, Gießen, Germany; b Laboratory of Biomechanics, Justus-Liebig-University Gießen, Gießen, Germany; c Adult Reconstruction, Vanderbilt University Medical Center, Nashville, TN, USA; d Laboratory for Experimental Radiology, Department of Diagnostic and Interventional Radiology, Justus-Liebig-University, Gießen, Germany; e Institute of Physical Chemistry, Justus-Liebig-University Giessen, Giessen, Germany; f Department of Orthopaedics and Trauma, Medical University Graz, Graz, Austria

**Keywords:** Aseptic loosening tibial arthroplasty, Cement viscosity and intrusion, Application pressure cement, Interface cement implant, Cement morphology knee arthroplasty, Micromotion tibial tray

## Abstract

**Background:**

Application time and viscosity are factors that can significantly affect the properties of bone cement and implant fixation. The aim of this study was to investigate the influence of different application times of 2 different cements on mechanical parameters, cement interdigitation, and cement distribution.

**Methods:**

P.F.C. Sigma tibial trays were cemented with high-viscous Palacos R and medium- to low-viscous Simplex P in an open-cell model. The application was performed at different times within the manufacturer’s specifications. Cement interdigitation and micromotion were measured with computed tomography scan using a novel method.

**Results:**

Significant differences of insertion forces were found at all times of cement application. Cement penetration decreased with increasing pressure and viscosity. No significant differences were shown for micromotion between Palacos R and Simplex P except for an increase for Simplex P from 3 to 7 minutes at the bone-cement interface. Simplex P appeared to trap air at the implant-cement interface at 3 minutes and increased at 7 minutes.

**Conclusions:**

Cement distribution and intrusion of Palacos R and Simplex P decreased with time. Simplex P trapped air at the implant-cement interface, decreasing the amount of contact at the implant-cement interface, which is worrisome for long-term implant fixation. Given the significant changes in cement properties after mixing, it is necessary for surgeons to understand the viscosity and timing of cement application to achieve optimal cement penetration and surface contact area to potentially decrease implant loosening. High-viscous Palacos R should be applicated immediately with doughing time and medium-viscous Simplex P for about 4 minutes considering a threshold of minimum pressure.

## Introduction

Aseptic loosening is a relevant cause for failure following primary cemented total knee arthroplasty. Implant fixation strength for cemented tibial implants appears to decrease with time, and loosening can occur at either interface (implant-cement or cement-bone) [1]. Failure at the implant-cement interface is common [2] and yet the mechanism of early failure remains unclear [3,4]. Mechanical properties of cement and its distribution appear to correlate with implant fixation. The interaction between mechanical properties of bone cement and viscosity has been proposed as a potential mechanism for aseptic loosening [1,5]. Micromotion at the implant-cement interface was noted to be reduced with increased interlocking depending on implant surface [6]. Regardless of the implant geometry and undersurface roughness, aspects of cement application are important to provide an optimal cement layer [[7], [8], [9], [10], [11]]. Optimizing cement technique has largely focused on cement penetration into bone. The influence of the primary distribution of the cement on the long-term fixation is not known [10,12]. The distribution and depth of cement penetration seem important for the fixation of the implant since the ideal volume of cement is not known [5,9,11,[13], [14], [15]]. The distribution of the cement is dependent on cement dough viscosity and force of application [3,16]. The components of cement influence its intrinsic properties, and the ideal cement viscosity for achieving long-term fixation remains unknown [5,7,17]. Furthermore, there remains minimal data to demonstrate how viscosity and application time can potentially change parameters associated with fixation.

Recently, an in vitro sawbone model was developed to study the cement fixation strength of tibial implants at the implant-cement and cement-bone interfaces [18] since polyurethane foam is a recognized model to study implant fixation [17]. The implantation force of 2 cements with low and high viscosity was recorded at different times of application, and measurement of relative micromotion at the cement interfaces was performed. It should be noted that micromotion appears to be a surrogate for initial implant fixation but does not necessarily represent the absolute fixation strength. To our knowledge, there is not a study that has evaluated the influence of different application times of 2 different cements on mechanical parameters, cement intrusion, and cement distribution of 2 commonly utilized bone cements. Therefore, we designed the following study to determine if cement viscosity and application time of 2 commonly utilized bone cements significantly change the distribution and volume of bone cement as well as the surface area of fixation at the implant-cement interface.

## Material and methods

The following study was performed utilizing an open-cell rigid foam model, which has previously been described [18].

### Bone model preparation

**Figure 1:**
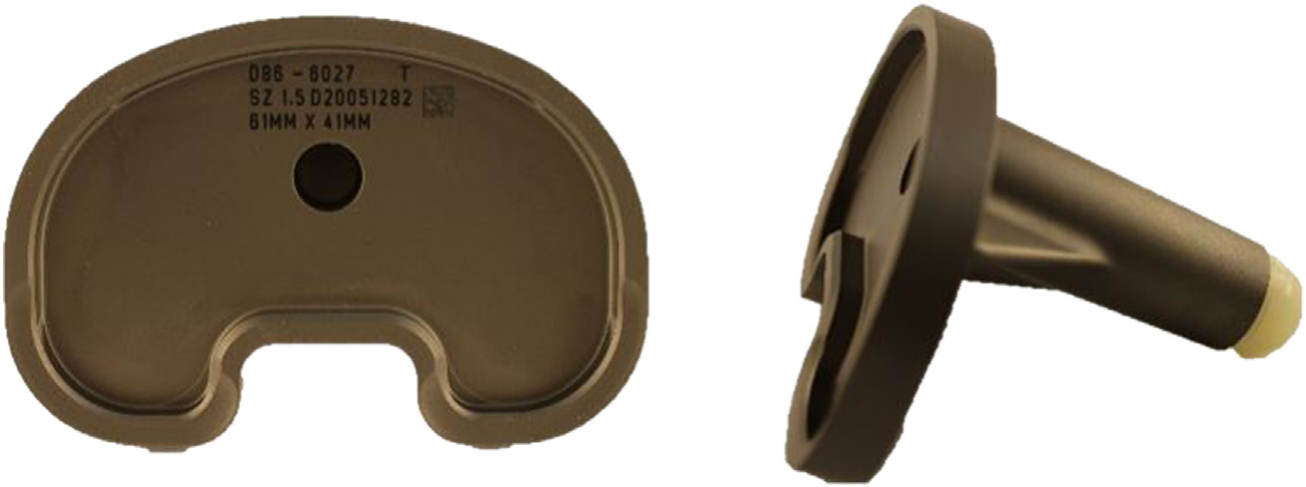
P.F.C. Sigma tibia component.

**Figure 2:**
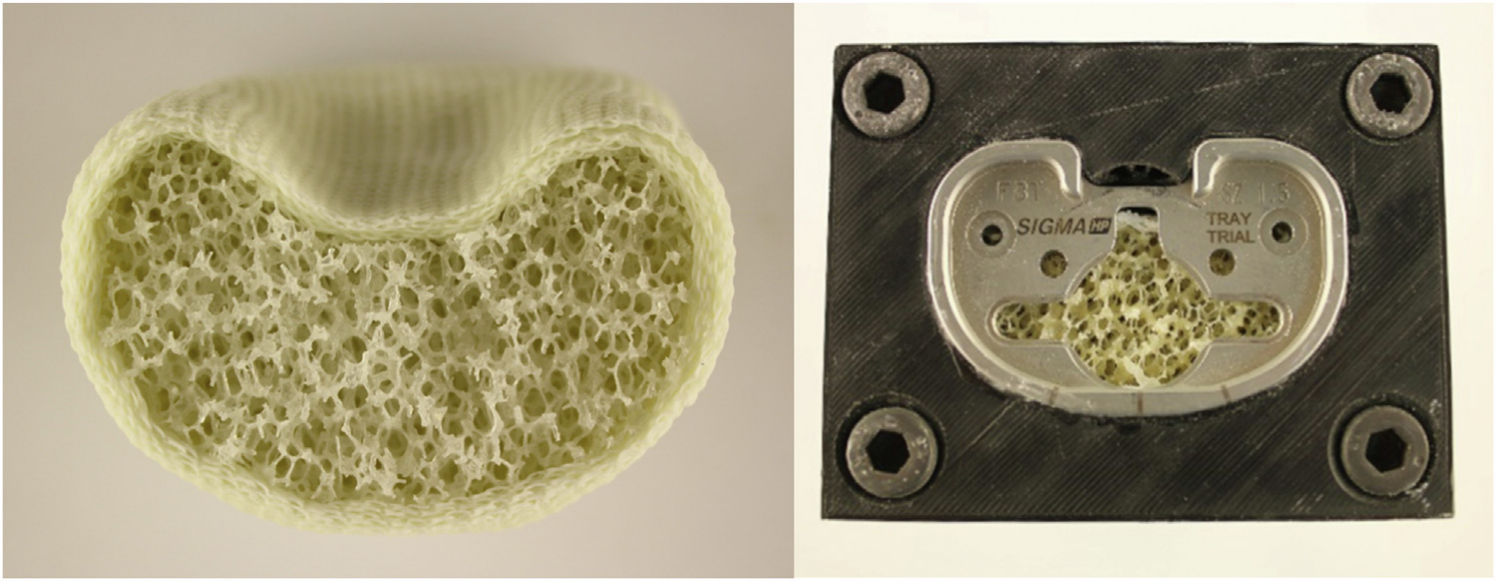
Open-pore bone model (left) and clamping of the bone model in the centering aid for the medullary cavity preparation (right).

**Figure 3:**
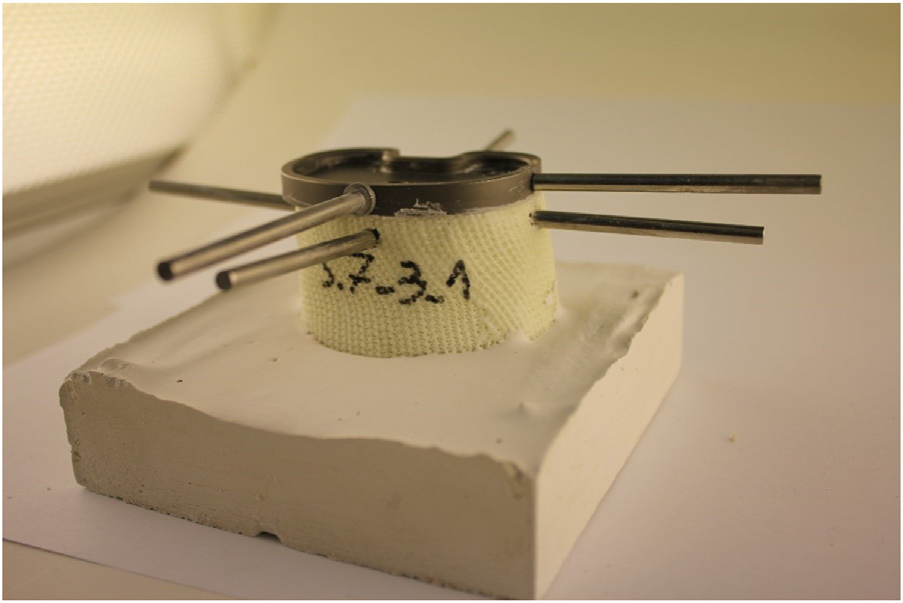
Bone model fixed in plaster including measuring pins.

**Figure 4:**
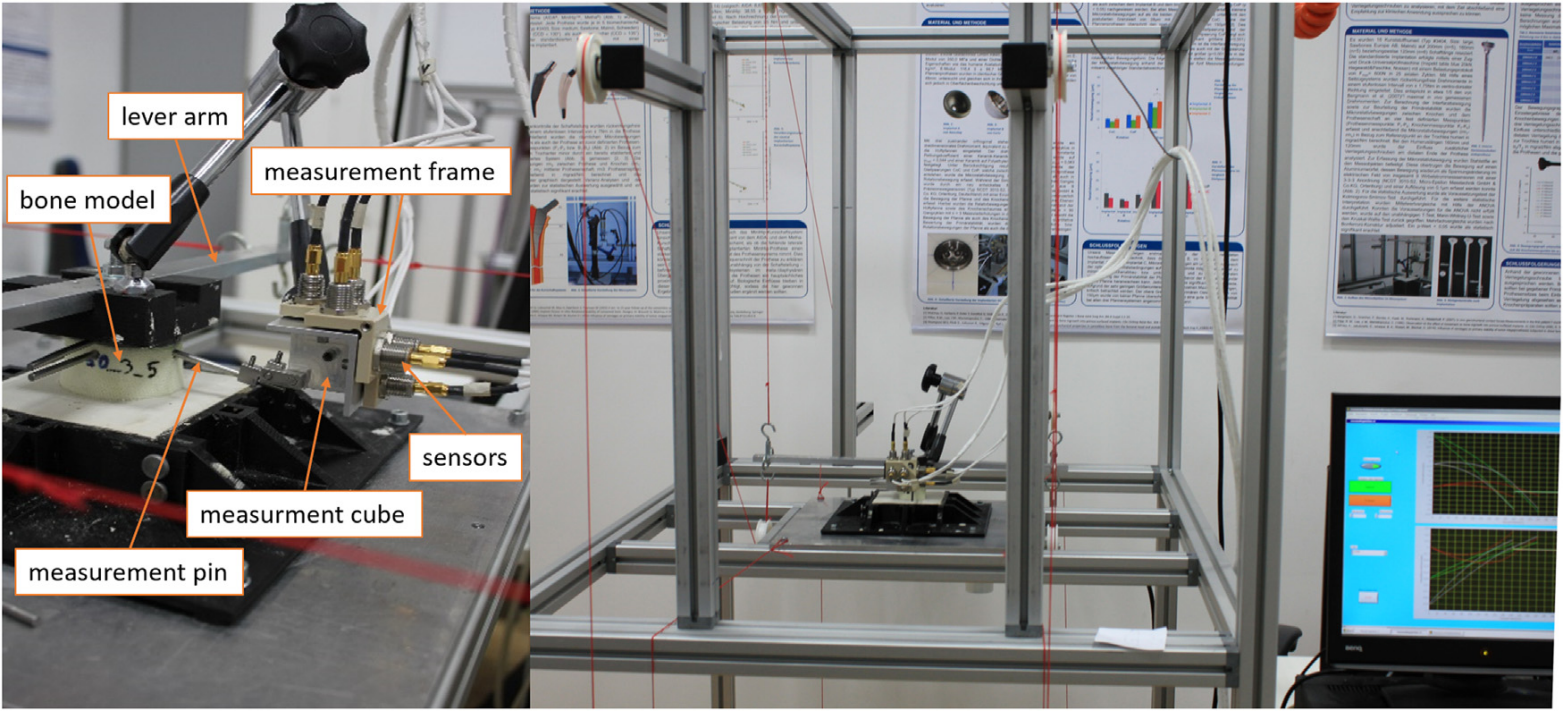
Measurement system (left) and entire measurement setup (right).

An open-cell rigid foam model fitting with the tibial tray was developed. The material properties of the sawbone model (Sawbone Europe AB, Malmö, Sweden) with a density of 20 pounds per cubic foot (density: 0.32 g/cc; porosity: 0.21; compressive strength: 1.3 MPa; Young´s modulus: 105 MPa) resemble human trabecular bone (density: 0.35 g/cc; porosity: 0.69 ± 0.1; elastic modulus: 116.4 ± 86.7 MPa) as much as possible, also considering the physiological open-cell structure [17,19]. The bone was milled and impacted with the original instruments of the manufacturer, additionally using a centering device, and then a size 1.5 P.F.C. Sigma (DePuy Synthes, Warsaw, IN, USA) tibial tray (Fig. 1) was implanted (Figure 2, Figure 3, Figure 4) [18].

### Bone cement

For cement application, the implants were cemented with either a high-viscosity bone cement, Palacos R (Heraeus Medical GmbH, Wehrheim, Germany), or a medium- to low-viscous cement, Simplex P (Stryker Howmedica Osteonics, Mahwah, NJ). Palacos R reaches a back pressure of 0.15 N within 15 seconds, and Simplex P approaches that point at 275 seconds [20]. Both cements were mixed under standardized conditions with vacuum and Palamix (Heraeus Medical GmbH, Wehrheim, Germany) for 30 seconds at constant room temperature of 19°C ± 1°C [18].

### Implantation procedure

A universal testing machine (Inspekt table blue 20kN, Hegewald & Peschke, Nossen, Germany) ensured standardized implantation. Each group consisted of n = 5 tibial trays. Each tibial tray was aligned with the model, lifted 60 mm, and cement was retrogradely applied into the medullary canal. The tibial plateaus were also loaded with cement using the cement gun and cement was applied manually to the implant surface, starting at various times: 3, 5, and 7 minutes for Simplex P and 3 and 5 minutes for Palacos R. (Table 1). No additional pressurizing was applied. A constant feed rate of 300 mm/min was maintained during implantation until reaching 0.1 mm from the bone, with continuous force measurement [18].

**Table 1:** Study design.

Bone density	Cement	Application time	N
20 PCF	Palacos R	3 min	5
		5 min	5
	Simplex P	3 min	5
		5 min	5
		7 min	5

PCF, pounds per cubic foot.

### Computed tomography examination

Following cement curing, a computed tomography scan was conducted with a 0.3 mm slice thickness using Siemens Somatom Force. Analysis was performed using Data viewer V.1.5.6.2 (Bruker microCT, Billerica, MA). Various parameters were measured, including total volume of binarized elements (Obj.V), the percentage of higher density elements relative to the total object (Obj.V/TV), complete surface area of connected higher density voxels (Obj.S), average 2D surfaces of connected higher density voxels (Obj.Pm), and 3D air volumes enclosed by higher density structures (Po(cl)%). Cement penetration within the bone structure was examined using the INFINITY internal clinic platform, with measurements taken at reproducible locations in the anterior (A), distal (D), lateral (B), medial (C), and 4 areas around the prosthesis stem (Pa, Pl, Pm, and Pd) based on the tibial tray's midline [18].

### Primary stability analysis

Nonreactive torques within a nondestructive range of ±3.5 Nm were applied to the cemented tibial tray and the model to assess stability. Multiple measurement pins (Fig. 3) were employed to monitor relative micromotion between the tibial tray and cement, the cement and bone, and the implant and bone. Bone measurement pins (B1/B2) were secured to the cortical bone dorsally and medially, positioned 5 mm below the implant edge using superglue. Ventral and lateral drilling, followed by drilling into the cement with a 1.8 mm drill, was done to fix the measurement pins (C1/C2) within the cement. For the implant, a 1.9-mm drill was used ventrally and medially to secure measurement pins (P1/P2) (Fig. 3) [18].

Relative micromotion of the implant, cement, and bone was measured using a contactless eddy current method [21] (Type NCDT 3010-S2, Micro-Epsilon, Ortenburg, Germany), offering a sensor resolution of 0.1 μm. An orthogonal measurement cube, made of aluminum, was attached to each measurement pin. This cube's movements were detected by 9 eddy current measurement sensors fixed in a measurement frame in a 3-3-3 arrangement [18].

The measurement frame was connected to the force-applying lever arm through a tripod arm, serving as a reference point. Torques were applied along the Z-axis, and tilting moments in extension/flexion and varus/valgus were transmitted, enabling the recording of relative micromotions (Fig. 4) [18].

### Data processing

The relative micromotions were analyzed using MATLAB (Version 2020b, The MathWorks, Inc., Natick, MA) to calculate the standardized angle of rotation α in mdeg/Nm. The data of the implantation force, relative micromotions, and the radiological evaluation are presented as mean values with an associated standard deviation. Statistical analysis was performed using SPSS 29.0 (SPSS Inc., IBM, Chicago, IL). ANOVA using the least significant difference post hoc test with logarithmized original values was performed for normally distributed data, and non-normally distributed data were analyzed with Kruskal-Wallis one-factor analysis of variance. Related to multiple testing procedures in pairwise comparison, *P*-values were adjusted according to Bonferroni. A *P*-value <.05 was considered statistically significant.

## Results

### Mechanical parameters

**Figure 5:**
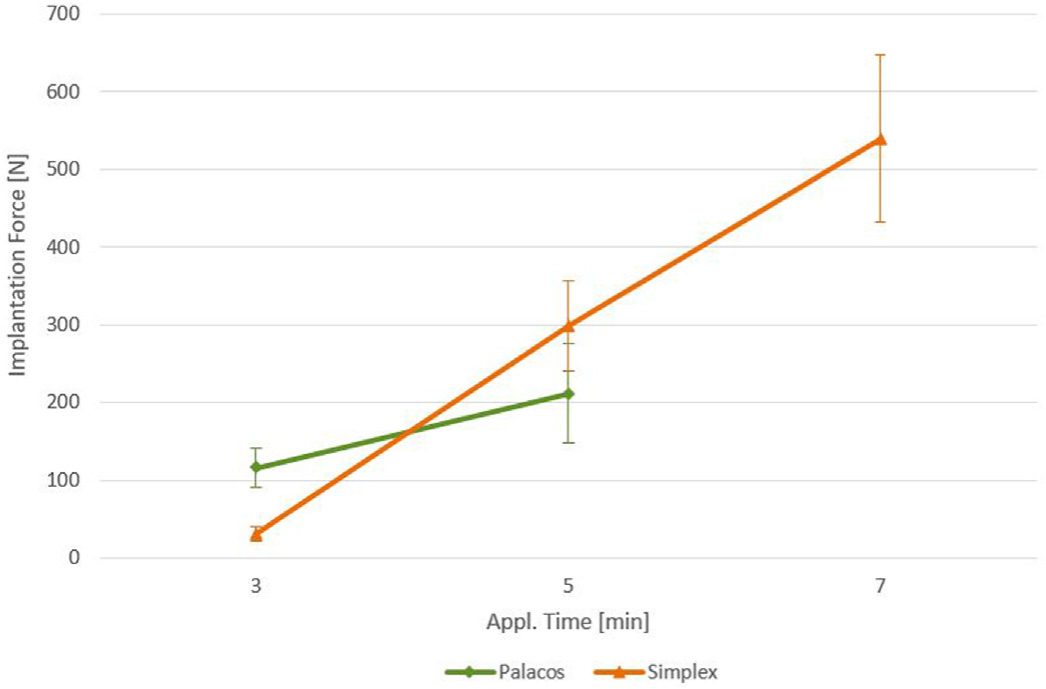
Implantation force taking into account the cement used and the application time.

Implantation force increased with the time of cement application, and there were significant differences between application times. Primary stability and relative micromotions did not significantly vary with the application time or cement type, except for Simplex P at 3 and 7 minutes at the cement-bone interface (Fig. 5). With prolonged application since mixing, Simplex P showed a tendency of increased micromotion with extension/flexion and varus/valgus force, and Palacos R showed higher values for extension/flexion force at 3 compared with 5 minutes (Table 2). In comparison, micromotion appeared small for both cements regarding rotational force, and no time-related effect was apparent (Table 2).

**Table 2:** Recorded mechanical parameters.

Mechanical parameters											
Cement	Application time	Implantation force [N]	Rotation (Z-axis) [mdeg/Nm]			Varus-valgus (X-axis) [mdeg/Nm]			Extension-flexion (Y-axis) [mdeg/Nm]		
			Cement-prosthesis	Cement-bone	Prosthesis-bone	Cement-prosthesis	Cement-bone	Prosthesis-bone	Cement-prosthesis	Cement-bone	Prosthesis-bone
Palacos	3 min	116.0 ± 25.6^a,c^	5.3 ± 3.1	3.3 ± 2.5	5.3 ± 4.3	4.1 ± 1.4	7.3 ± 7.3	5.8 ± 6.6	12.5 ± 24.7	18.8 ± 9.4	15.1 ± 12.1
Palacos	5 min	211.8 ± 63.7^b,c^	2.2 ± 1.5	2.8 ± 2.9	4.8 ± 3.4	5.9 ± 4.7	9.5 ± 7.0	4.5 ± 2.5	1.9 ± 1.5	8.6 ± 7.3	7.5 ± 7.6
Simplex	3 min	31.4 ± 9.0^a,d,e^	2.9 ± 2.0	4.3 ± 2.6	5.5 ± 3.8	7.4 ± 5.4	12.6 ± 11.9^a^	14.8 ± 11.7	4.5 ± 2.5	24.0 ± 10.0	24.4 ± 14.5
Simplex	5 min	298.8 ± 57.9^b,d,f^	3.7 ± 1.8	2.8 ± 1.2	5.0 ± 3.3	10.1 ± 15.1	6.0 ± 9.4	10.3 ± 13.6	1.4 ± 2.1	16.5 ± 17.4	15.7 ± 16.3
Simplex	7 min	540.0 ± 107.2^e,f^	3.9 ± 3.2	5.7 ± 2.6	9.6 ± 1.9	12.2 ± 5.2	19.7 ± 14.0^a^	25.4 ± 6.7	2.5 ± 3.6	37.8 ± 24.7	36.5 ± 24.0
*P*-values		a < .001; b = .032; c = .002; d < .001; e < .001; f = .004	-	-	-	-	a = .034	-	-	-	-

Small superscript letters indicate significant *P*-values in pairwise comparison.

### Radiology analysis

The time of cement application significantly influenced the volume of both cement types. All radiologic parameters changed with application time. Air collection under the implant surface increased for Simplex P from 3 to 7 minutes. Higher cement volume at 5 minutes and lower volume at 3 and 5 minutes compared to 7 minutes were found. Cement penetration depth (intrusion) varied, with Palacos R having higher penetration in the anterior area at 3 minutes and Simplex P showing higher penetration at 5 and 7 minutes. Cement mantle volume was significantly decreased with increased application time and viscosity for both cements. The cement surface did not significantly differ at 3 and 5 minutes; however, there was a significant decrease in Simplex P at 7 minutes (Table 3).

**Table 3:** Cement properties based on radiological parameters.

Cement distribution						
Cement	Application time	Obj.V [mm³]	Obj.V/TV [%]	Obj.S [mm^2^]	Obj.Pm [mm]	Po(cl)%. [%]
Palacos	3 min	308,316.2 ± 30,779.6^a^	61.5 ± 2.9	127,092.9 ± 8833.4	751.8 ± 44.6	19.7 ± 1.6
Palacos	5 min	288,187.2 ± 65,648.2	59.3 ± 5.8^a^	128,057.4 ± 13,448.3	745.7 ± 75.1	16.1 ± 7.4
Simplex	3 min	388,957.3 ± 13,556.0^a^	64.5 ± 3.5	137,681.2 ± 13,983.5^a^	760.6 ± 51.4^a^	15.0 ± 0.9
Simplex	5 min	329,530.2 ± 13,042.0	64.3 ± 2.3^a^	129,407.1 ± 8117.2^b^	756.2 ± 55.1^b^	18.8 ± 1.1
Simplex	7 min	284,023.9 ± 12,455.0	63.2 ± 1.3	108,440.4 ± 2101.6^a,b^	664.4 ± 12.5^a,b^	21.8 ± 1.6
*P*-values		a = .010	a = .032	a < .001; b = .007	a = .017; b = .023	-

Small superscript letters indicate significant *P*-values in pairwise comparison.

**Figure 6:**
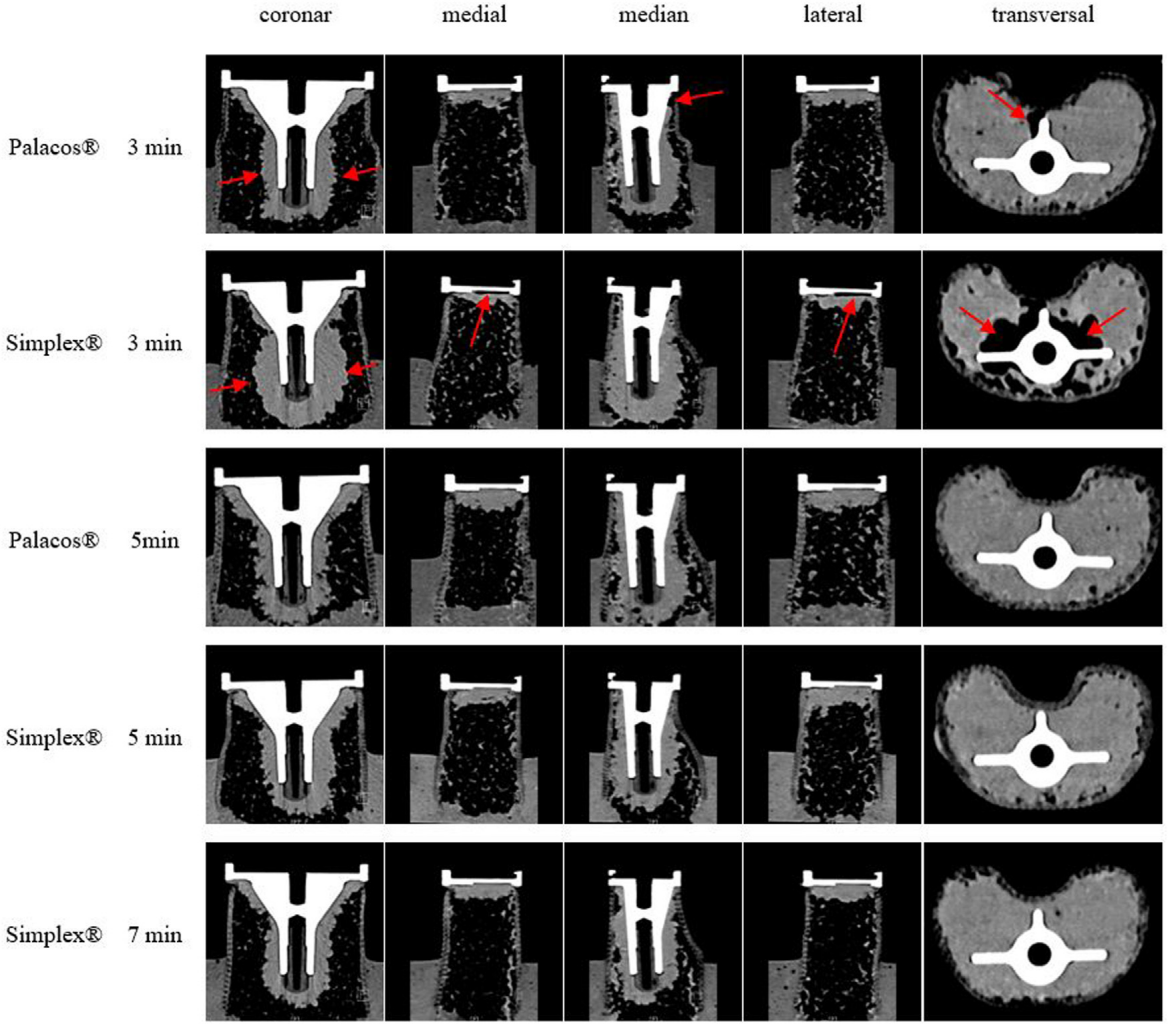
Cement penetration of the different cements considering the different application times at different levels.

Some cases had increased air entrapment under the tibial tray for Simplex P at 3 minutes, since no significant difference was found between Palacos R at 3 minutes and Simplex P at 5 minutes. A nonsignificant increase in air entrapment was noted for Simplex P from 3 to 7 minutes (Table 3). The cement distribution around the implant varied with application time, viscosity, and cement type, with Simplex P resembling an upside-down mushroom and Palacos R closely fitting the implant (Fig. 6).

## Discussion

The prevalence of tibial implant loosening varies in the literature [7], and there are a variety of factors that appear to alter implant fixation [5]. Some studies suggest that loosening is associated with a cement penetration depth of less than 2 mm intrusion [9]. Failures at the cement-bone interface might be improved with better cement penetration, but implant-cement interface failures remain a common cause of failure. For this reason, cement application to the tibial tray and tibial bone is recommended [15] and cementing the stem of tibial trays enhances primary stability [22]. Interestingly, there is no consensus on guidelines for the cementation technique in total knee replacements [11]. Therefore, we designed the following study to evaluate how cement viscosity and timing of cement application alter the insertion properties of bone cement.

Implantation force significantly varies across all application times for both high-viscous Palacos R and medium-viscous Simplex P. The timing of cement application appears to correlate with fixation strength [7], with early application of high-viscosity cement being beneficial. Notably, Grupp [17] found no significant differences in fixation strength among 5 different cement types using push-out test. We did not test implants to failure and cannot conclude differences in absolute fixation strength between bone cements and application times. We found that the applied cement volume decreases with increasing pressure, viscosity, and time. Similarly, we noted significant differences in implantation force between Palacos R and Simplex P at similar application times. For instance, at 3 minutes, the implantation force is significantly lower for Simplex P compared to Palacos R because of completely different viscosity of the dough. However, there was a substantial increase observed for Simplex P at 5 minutes, resulting in a comparable force to Palacos R at 3 minutes. There is likely a comparable implantation force for both cements around 160 N that occurs in around 4 minutes. This data supports that the cement viscosity between these 2 cements appears to converge within the 3- to 7-minute range. Additionally, this data suggests that earlier application of bone cement increases cement volume.

As stated per definition in the International Organization for Standardization and American Society for Testing and Materials standards, both cements are characterized by a comparable dough viscosity and intrusion at completely different times: the doughing time for Palacos R is about 90 seconds and for Simplex P is 3.5 minutes. The long mixing and waiting time for Simplex R might have disadvantages in clinical practice [20,23]. Using low-viscosity cement bears the risk of insufficient pressure generation needed for uniform cement distribution. A certain viscosity is required to achieve the desired penetration depth and morphology of the cement around the implant while avoiding excessive cement extrusion during implantation. The interaction of pressure and viscosity is crucial for the cement-implant connection. Prolonged application times should be avoided [20], as increased implantation pressure negatively affects cement distribution and structure. The ideal implantation force for optimal fixation at the implant-cement and cement-bone interfaces appears to be influenced by the type of cement, viscosity, and the time elapsed since application.

While it was noted that cement volume appears to decrease with increasing time and viscosity, this was not the only worrisome finding with delayed cement application times. It was noted that fixation of cement declines for Simplex P between 3 and 5 to 7 minutes. During this time, air bubbles appear to disrupt the implant-cement interface leading to a decrease in contact surface area. Therefore, Simplex P ideally should not be applied too early since International Organization for Standardization doughing time of Palacos R is about 90 seconds and for Simplex P about 3.5 minutes. Our results suggest the best results for application of Simplex P between 4 and 6 minutes to prevent disruption from air bubbles. There was no significant difference in surface area between Simplex P and Palacos R if both cements had comparable viscosity dough. Increased insertion pressure beyond 5 minutes of Simplex P application fails to prevent a significant decrease in contact surface at 7 minutes. The significantly higher applied volume of Simplex P compared with Palacos R at 3 minutes was not correlated with an increase in contact surface since this appeared comparable for both cements at that time (Table 3).

In a human cadaver model, the strength of fixation at the cement-bone interface was found to be higher for standard viscosity cement than for lower viscosity cement, suggesting better intrusion or interdigitation [13] and early cement application and cementing the keel of the tibial implant increase fixation strength for Palacos R more than Simplex P [7]. The strength of the implant-cement interface does not steadily increase with greater cement penetration depth [24,25]. Therefore, the applied cement volume should be restricted to the improvement of fixation strength since the risk of using bone cement too early might be associated with higher risk of embolism and necrosis, and blood could more easily mix with the dough [23]. Distal cement flow reduces proximal pressure [26] which might bear a risk for occasional air entrapment below the tibial tray during early application of Simplex P at 3 minutes. The fixation of Simplex P significantly changes with application time, emphasizing that low-viscosity cement should not be applied too early (Table 2, Table 3, Table 4).

**Table 4:** Measurement results of the cement penetration depth.

Cement penetration [mm]									
Cement	Application time	Plateau				Stem			
		Anterior	Lateral	Medial	Distal	Anterior (P1)	Lateral (P2)	Dorsal (P3)	Medial (P4)
Palacos	3 min	18.0 ± 15.4^a^	7.2 ± 1.5^a^	7.0 ± 1.6^a^	5.5 ± 1.8^a^	6.3 ± 2.3^a^	6.4 ± 1.6^a,b^	8.1 ± 1.1	8.4 ± 1.3
Palacos	5 min	37.0 ± 16.7	6.7 ± 0.7	6.1 ± 1.1	5.5 ± 1.8	7.9 ± 1.0	8.3 ± 0.8^b^	8.4 ± 1.1	6.9 ± 2.2
Simplex	3 min	8.1 ± 12.9^a,b,c^	5.4 ± 3.0^a,b^	4.9 ± 0.4^a^	10.0 ± 1.8^a,b^	7.9 ± 0.7^a^	11.8 ± 0.9^a,c,d^	10.0 ± 0.6a,b	10.5 ± 0.9^a,b^
Simplex	5 min	37.7 ± 18.9^b^	5.8 ± 0.5^b,c^	6.9 ± 0.6	7.0 ± 1.4	8.2 ± 0.6	8.7 ± 1.5^c,e^	7.2 ± 2.1a	5.9 ± 1.1^a^
Simplex	7 min	36.2 ± 15.8^c^	6.6 ± 0.7^c^	6.9 ± 0.7	6.5 ± 0.5^b^	7.2 ± 1.5	6.4 ± 0.8^d,e^	6.1 ± 0.5b	5.5 ± 1.5^b^
*P*-values		a = .038; b = .008; c = .006	a = .037; b = .006; c = .006	a = .002	a < .001; b = .047	a = .048	a < .001; b = .015; c = .032; d < .001; e = .032	a = .015; b < .001	a = .005; b = .002

Small superscript letters indicate significant *P*-values in pairwise comparison.

Cement penetration into the bone of 3-4 mm is considered ideal, with greater penetration achieved in the dough phase compared to the liquid phase [14] since a cement mantle of at least 1.1 mm below the tibial tray is favored to prevent failure at the cement-bone interface [4]. Certain regions, like the posterior condyles, are more prone to poor cementation [27], and differences in cement penetration at various areas are evident. Variations of the cement mantle at the interface may affect gap balance, with 3-5 mm of intrusion recommended for ideal cement interdigitation into bone [24]. Therefore, a homogenous intrusion of cement with a desired depth [4,14] without excessive applied volume considering the individual properties of the cement seems favorable for fixation and prevention of negative impacts [23].

A homogeneous cement distribution requires a certain pressure and viscosity to prevent air entrapment. In the early application phase, Palacos R forms a tight connection with the implant, whereas Simplex P exhibits a wider distribution with less interconnectivity. Excessive cement application may not necessarily enhance fixation strength, and surface area is not significantly correlated with this parameter (Table 2, Table 3, Table 4). Failure of tibial implants is more frequent at the interface between the implant and cement [28,29]. The volume of cement below the tibial tray decreases with increasing distance from the implant, and the distribution differs by tibia quadrant [30]. The volume of applied cement decreases with increasing application times for both cements, with significantly higher volume observed for Simplex P at 3 minutes. Therefore, individual application time of both cements, considering different viscosity of the cement appear to be beneficial in improving cement-bone fixation, potentially with an increased volume of bone fixation and through the prevention of entrapped air, decreasing fixation at the implant-cement interface. An ideal combination of pressure and viscosity seems important for both cements to create the best fixation of the implant.

For both cements, there were no significant differences in micromotion between the implant-cement and cement-bone interfaces, despite differences in cement penetration (Table 2, Table 3, Table 4). Micromotion also does not significantly differ between Palacos R and Simplex P; all values fall within a clinically stable range of movement. However, there is a significant difference in micromotion between 3 and 7 minutes of Simplex P application at the cement-bone interface under varus-valgus stress. Small variations in measurements do not suggest a significant difference in primary fixation strength at the tibial tray-cement interface, and there is no correlation between micromotion and cement distribution in different areas. The significantly higher impact strength and toughness of Palacos R compared to Simplex P are likely related to its significantly higher molecular weight [31]. Fatigue testing of cement may reveal differences in mechanical parameters not apparent under static conditions [17]. There is no universal limit for micromotion of fixation of implants, as a wide range appears stable [32,33].

The long-term impact of cement degradation on these primary micromotion differences remains unclear [1,4]. It is essential to consider tibial implant design in response to varying stress conditions, such as varus-valgus and flexion-extension, as well as prolonged cement application. Increased impaction force at 7 minutes with Simplex P may not enhance the implant-cement interface's fixation due to changes in cement interdigitation caused by rising viscosity and increased air entrapment.

While this study represents one of the first attempts to correlate cement viscosity and application time with implant fixation and cement distribution, there are several limitations that may affect the generalizability of our conclusions. The model used in our experiments does not perfectly mimic the clinical scenario, and this nonphysiologic approach may limit the applicability of our findings to real-world conditions. The variability in temperature and humidity during cementation, which can significantly affect the curing time, was not accounted for in our study and should be considered in future research. Additionally, the use of pressure as a variable may not fully capture the clinical variability seen in practice. The observed inflection points in viscosity, particularly with respect to Simplex P, suggest that further investigation into the complexity of cement hardening and its impact on fixation is needed. We caution against interpreting our findings as a comprehensive guide to cementing without considering other critical variables such as operating room conditions and mixing parameters. These factors can significantly influence outcomes and should be carefully evaluated in clinical practice.

This study was performed in an open-cell model and therefore has some inherent design limitations. The model likely represents a “best-case” cement technique as there was no lipid or blood contamination present and motion was limited by the insertion instrument. Only one implant and 2 bone cements were utilized, which potentially limits the generalizability of the study. The 20 pounds per cubic foot open-cell Sawbone model is even more porous than the similar closed-cell Sawbone both closely resembling properties of human cancellous bone [[17], [18], [19]] since open-cell structure is physiological. The constant feed rate was chosen to ensure reproducible values for both the implantation time and the gap between the tibial model and the implant, as well as allowing accurate capture of the force values generated during implantation. We are aware that this work step of implantation does not correspond to the clinical situation but provides a good reproducibility allowing statistical comparison of the values in our in vitro model. We chose 3 time points for cement application for Simplex P and 2 for Palacos R. While more time points might have allowed for even more precise examination for descriptive comparisons, we believe the time points we have chosen help tremendously in educating surgeons that earlier cement application might be beneficial.

## Conclusions

Timing of cementation might influence effectiveness of fixation since implantation force increases with prolonged application time of both Palacos R and Simplex P cement. Simplex P showed a significantly lower initial implantation force but was noted to increase much quicker than Palacos R. While micromotion at the cement interfaces did not reveal a significant difference between both cements at any time of application.

Palacos R and Simplex P showed comparable distribution and intrusion behavior within their individual application time since viscosity of Simplex P changed more rapidly. Early application of Simplex P favors emergence of larger volumes of cement neither increasing the surface area at the interfaces nor improving mechanical strength. Late application of Simplex P might reduce cement penetration and contact surface while also increasing air entrapment. Respecting application time comparable fixation strength should be obtained with both cements. A pressure threshold and an ideal interaction with cement viscosity appear necessary for ideal interdigitation and intrusion. Palacos R reveals less change in viscosity during a shorter recommended application period than Simplex P.

## Funding

The study was supported by 10.13039/100008127DePuy Synthes, Umkirch, Germany; 10.13039/100030856Heraeus Medical, Yardley, PA, USA; and 10.13039/100030856Heraeus Medical, Wehrheim, Germany.

## Conflicts of interest

J. R. Martin is a paid consultant for DePuy. A. Jahnke receives research support from DePuy and receives financial support from Heraeus GmbH. C. A. F. Ulloa receives research support from Heraeus Medical GmbH and receives financial support from DePuy. C. Glameanu is a paid consultant for DePuy. C. Fölsch receives research support from Heraeus and receives financial support from DePuy Synthes. C. Paul and K.-D. Kühn receive research support from Heraeus GmbH. All other authors declare no potential conflicts of interest.

For full disclosure statements refer to https://doi.org/10.1016/j.artd.2024.101476.

## CRediT authorship contribution statement

**Christian Fölsch:** Writing – review & editing, Writing – original draft, Supervision, Project administration, Methodology, Funding acquisition, Conceptualization. **Julia Schirmer:** Investigation, Data curation. **Cosmin Glameanu:** Investigation, Data curation. **Bernd Ishaque:** Writing – review & editing. **Carlos Alfonso Fonseca Ulloa:** Writing – review & editing, Supervision, Software, Methodology, Investigation, Formal analysis, Data curation. **Torben Harz:** Software, Formal analysis, Data curation. **Markus Rickert:** Writing – review & editing, Resources, Project administration, Funding acquisition. **John Ryan Martin:** Writing – review & editing, Supervision, Formal analysis, Conceptualization. **Jan Scherberich:** Methodology, Formal analysis, Data curation. **Jessica Steinbart:** Methodology, Formal analysis, Data curation. **Gabriele Krombach:** Writing – review & editing, Resources, Methodology. **Christian Paul:** Resources, Methodology, Data curation. **Klaus-Dieter Kühn:** Writing – review & editing, Methodology, Formal analysis, Conceptualization. **Alexander Jahnke:** Writing – review & editing, Writing – original draft, Supervision, Resources, Project administration, Methodology, Funding acquisition, Formal analysis, Data curation, Conceptualization.

## Appendix A Supplementary data

Conflict of Interest Statement for Fonseca Ulloa

Conflict of Interest Statement for Glameanu

Conflict of Interest Statement for Harz

Conflict of Interest Statement for Ishaque

Conflict of Interest Statement for Jahnke

Conflict of Interest Statement for Krombach

Conflict of Interest Statement for Martin

Conflict of Interest Statement for Paul

Conflict of Interest Statement for Rickert

Conflict of Interest Statement for Schirmer

Conflict of Interest Statement for Kühn

Conflict of Interest Statement for Fölsch

Conflict of Interest Statement for Scherberich

Conflict of Interest Statement for Steinbart
